# Supplementary material for: Nucleotide-induced hyper-oligomerization inactivates transcription termination factor ρ
Source: Nat Commun. 2025 Feb 15;16:1653. doi: 10.1038/s41467-025-56824-8 (PMC11829017; doi:10.1038/s41467-025-56824-8)

Supplementary Information

Nucleotide-induced hyper-oligomerization inactivates transcription termination factor ρ

Bing Wang[1#], Nelly Said[2#], Tarek Hilal[2,3], Mark Finazzo[1], Markus C. Wahl[1,4*], Irina Artsimovitch[1*]

[1] Department of Microbiology and Center for RNA Biology, The Ohio State University, Columbus, OH, USA

[2] Freie Universität Berlin, Institute of Chemistry and Biochemistry, Laboratory of Structural Biochemistry, Takustr. 6, D-14195 Berlin, Germany

[3] Freie Universität Berlin, Institute of Chemistry and Biochemistry, Research Center of Electron Microscopy and Core Facility BioSupraMol, Fabeckstr. 36a, 14195 Berlin, Germany

[4] Helmholtz-Zentrum Berlin für Materialien und Energie, Macromolecular Crystallography, Albert-Einstein-Str. 15, D-12489 Berlin, Germany

[#] equal contribution

* Correspondence to: artsimovitch.1@osu.edu; markus.wahl@fu-berlin.de

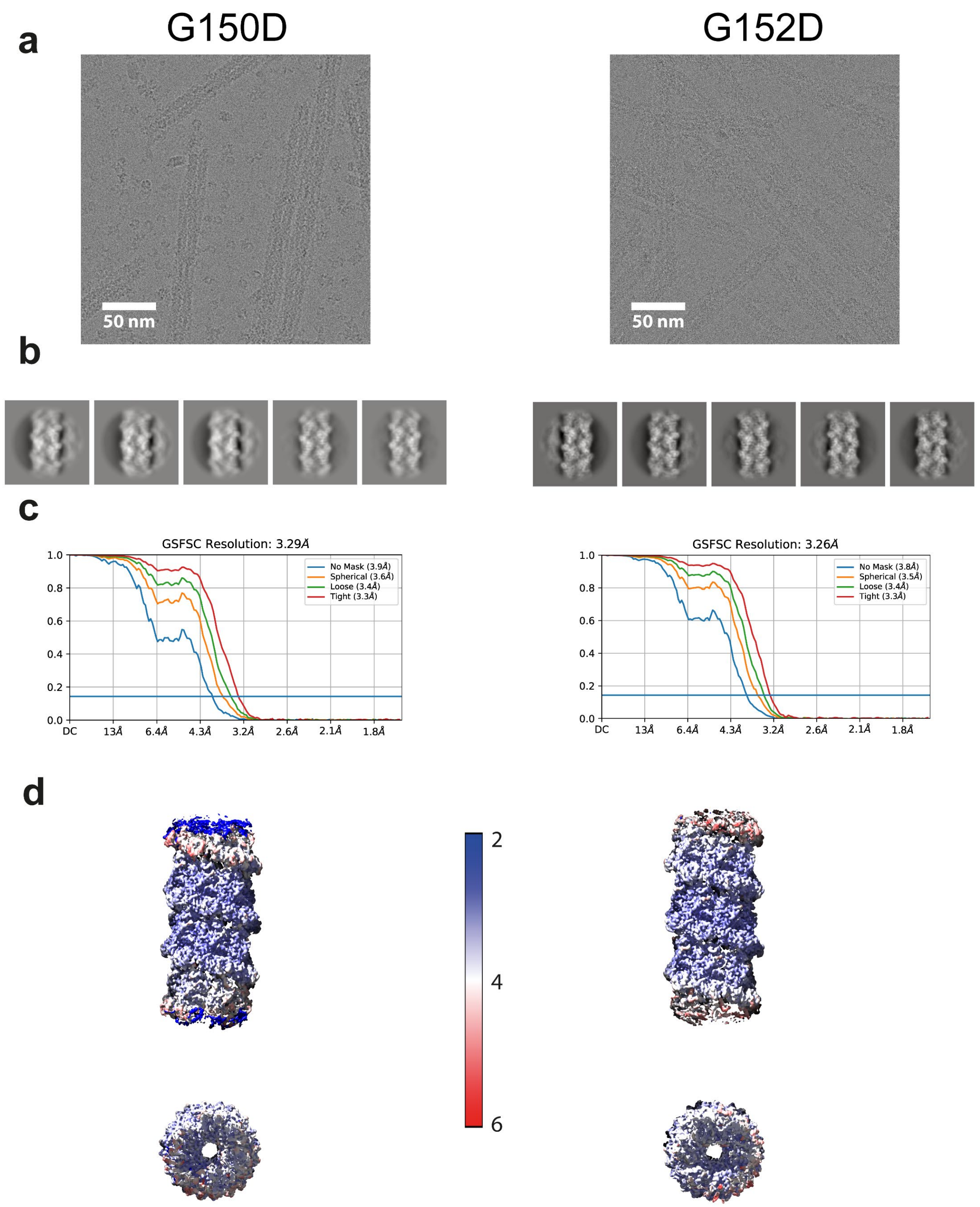

Supplementary Fig. 1. CryoEM analysis of ADP bound ρ variants G150D•H_8 (left) and G152D•H_8 (right). a, Representative cryoEM micrographs. Scale bars, 50 nm. Another G150D•H_8 ρ micrograph is shown in Fig. 1b. **b**, Selected 2D class averages after reference-free 2D classification. **c**, Gold standard Fourier shell correlation analysis after helix refinement. **d**, Side (top) and top (bottom) views of the 3D reconstructions, colored by local resolution.

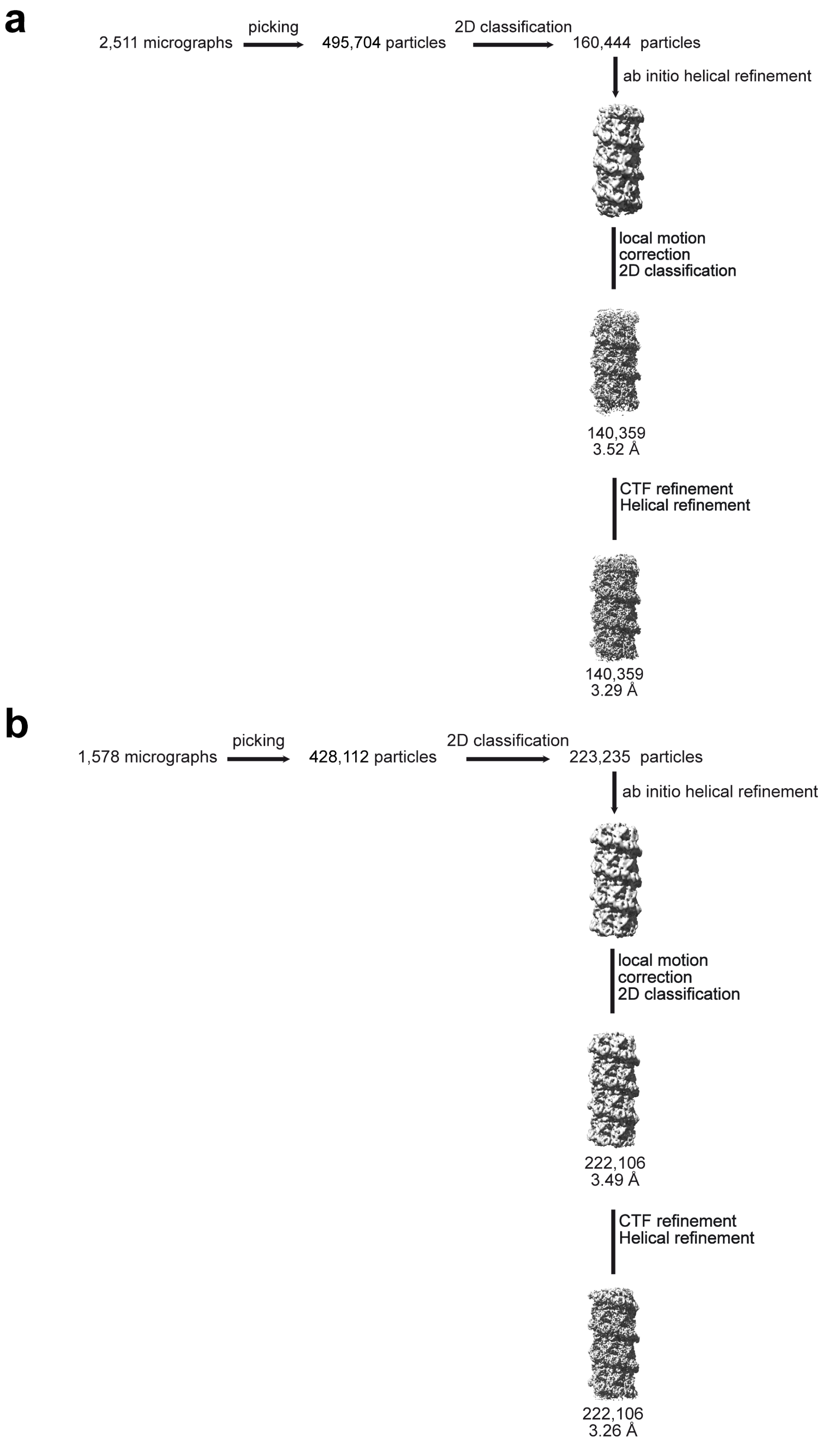

Supplementary Fig. 2. Sorting schemes of (a) G150D•H_8 filament and (b) G152D•H_8 filament.

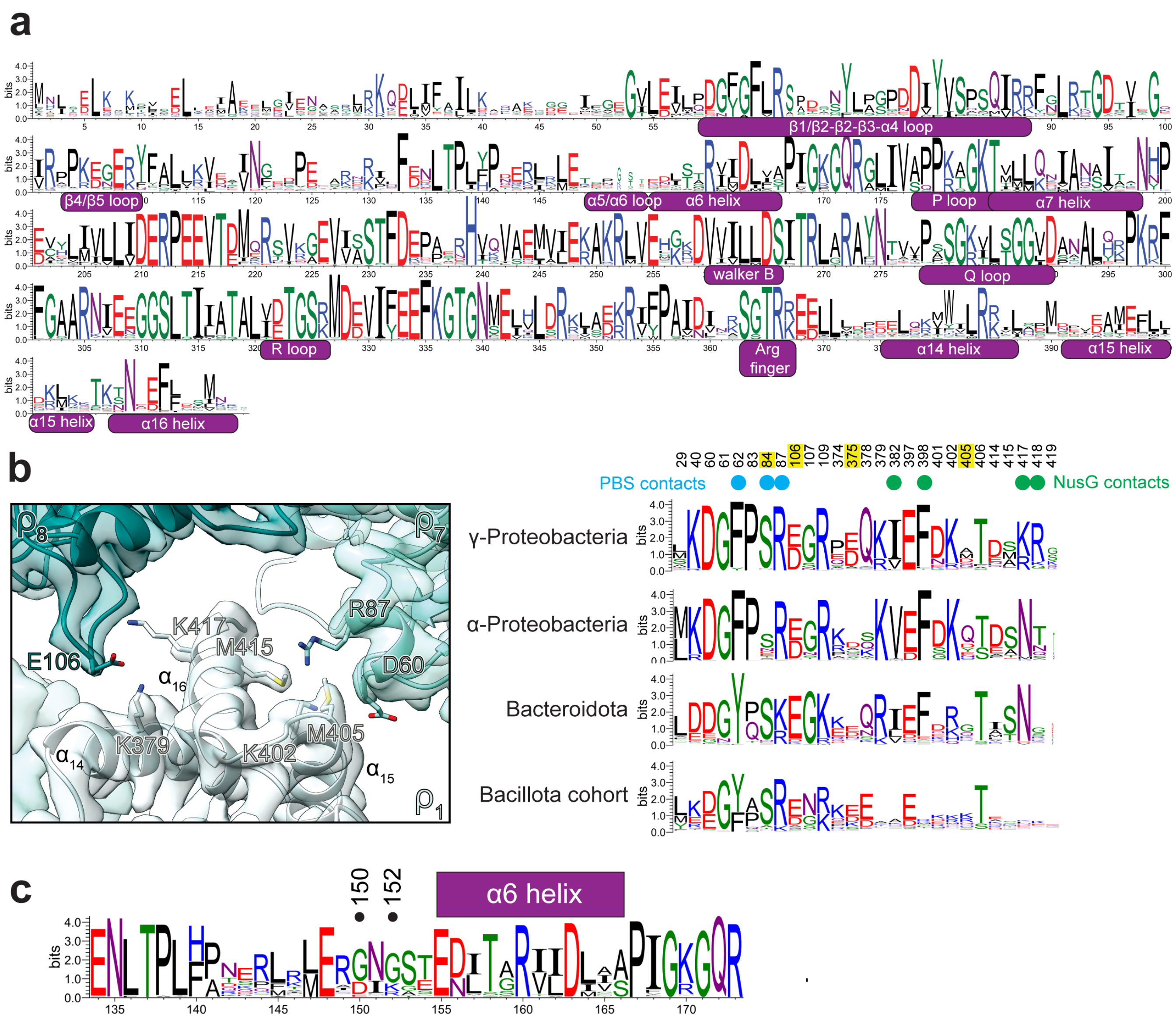

Supplementary Fig. 3. **Sequence conservation of ρ. a**, The sequence logo was generated from the alignment of ρ representatives from 42 phyla (Supplementary Data 1a). Residues are indexed according to *E. coli* ρ (NP_418230.1). The C-terminal domain, which contains key catalytic elements, exhibits higher conservation. **b**, Conservation of the filament interface. Left, structural view of the filament interface with experimental density shown as transparent surface. Close residues are labeled. Right, sequence logos showing the conservation of the filament interface. The four residues mutated to Cys are highlighted in yellow. PBS (primary RNA binding sites; cyan dots) and NusG (green dots) contact residues overlap with the filament interface. The Bacillota cohort includes phyla Bacillota, Bacillota-A, -B, -D, -E, -F, -G, and -I. **c,** Residue conservation around the connector and α6 helix in γ-Proteobacteria. Positions 150 and 152 are indicated.

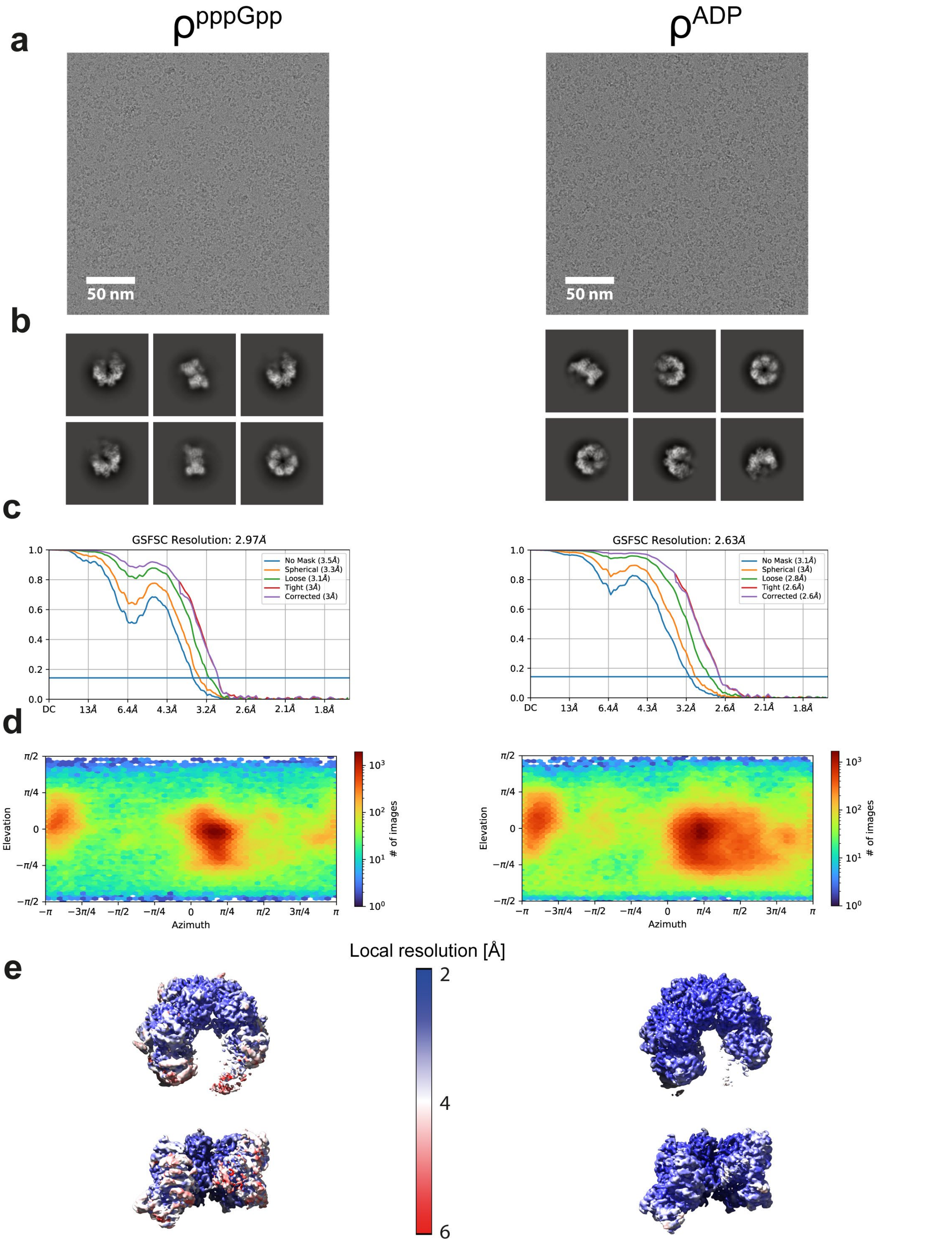

Supplementary Fig. 4. **CryoEM analysis of untagged ρ bound to pppGpp (left) or ADP (right). a**, Representative cryoEM micrographs. Scale bars, 50 nm. **b**, Selected 2D class averages after reference-free 2D classification. **c**, Gold standard Fourier shell correlation analysis after NU refinement. **d**, Viewing direction distribution after NU refinement. **e**, Top (top) and side (bottom) views of the 3D reconstructions, colored by local resolution.

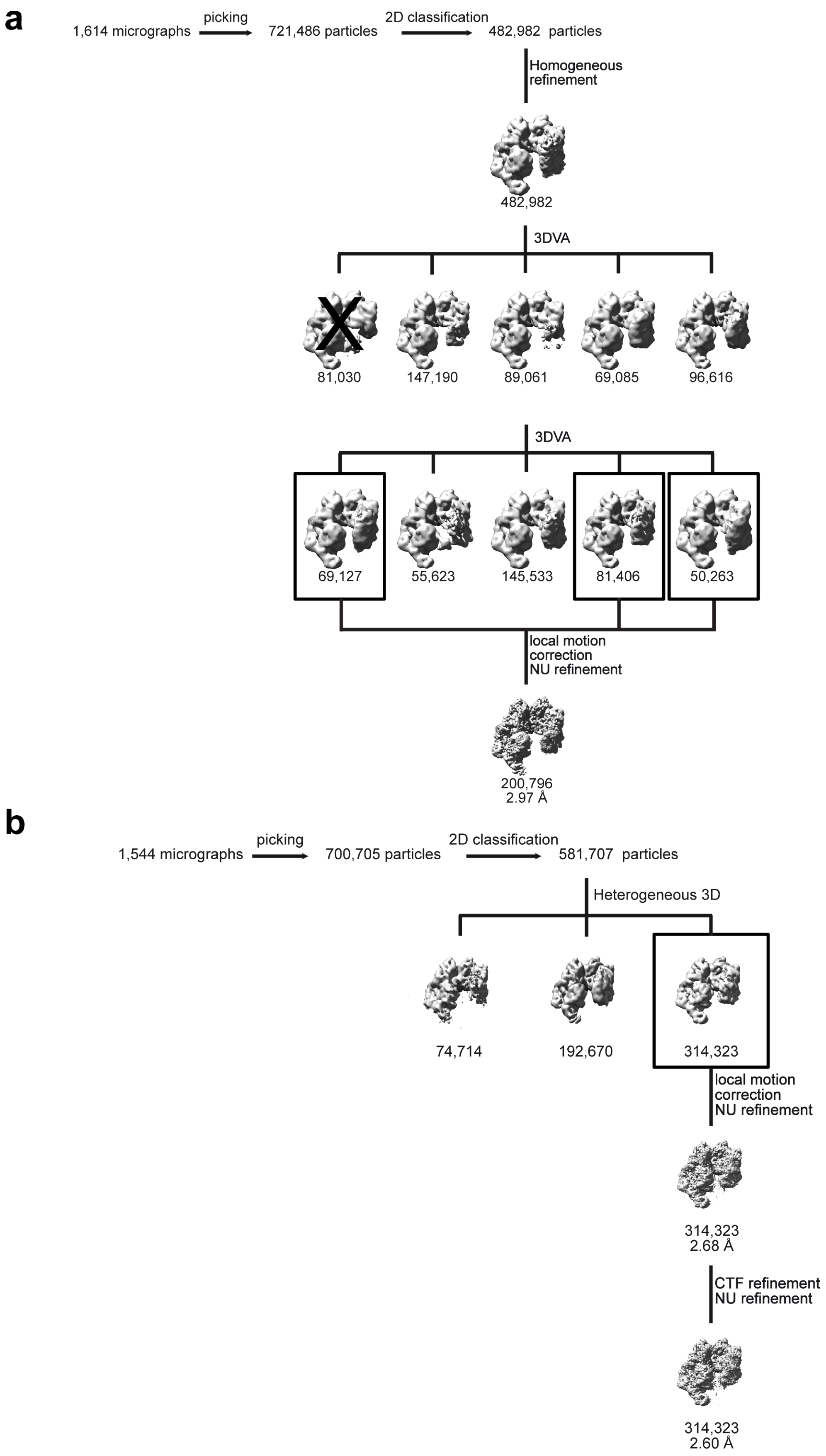

Supplementary Fig. 5. Sorting schemes of (a) untagged ρ bound to pppGpp or (b) ADP.

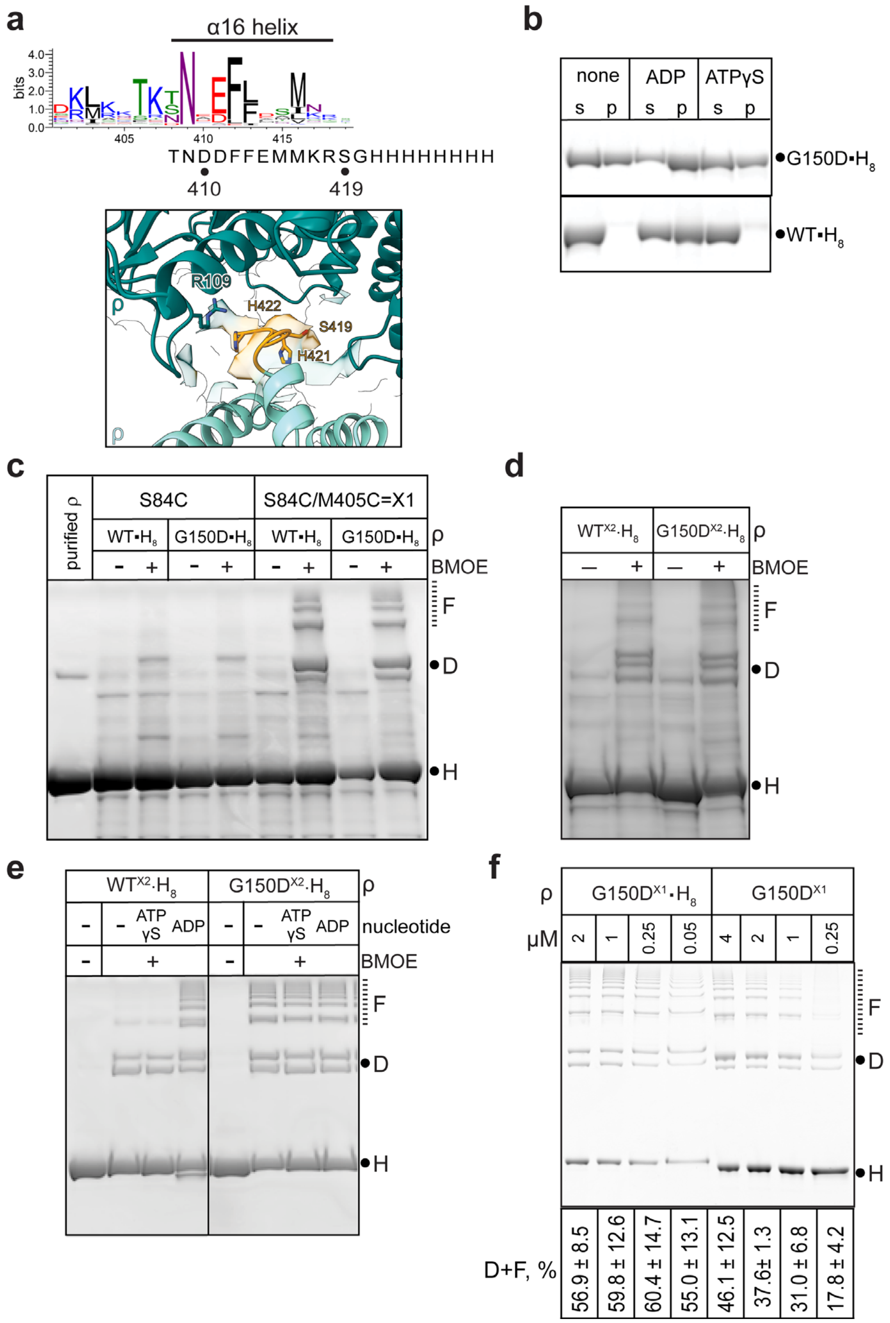

Supplementary Fig. 6. BMOE mediated *ex vivo* cysteine cross-linking. a, (top) Sequence logo showing the conservation of the ρ C-terminus across the bacterial kingdom. Residues forming α16 helix are indicated. (bottom) The H_8 tag added to the C-terminus of ρ (top) is located at the filament interface. **b**, Nucleotide-dependent polymerization of ρ. Indicated C-terminally H_8-tagged ρ variants (1 µM) were incubated with 2 mM nucleotides and centrifuged at 110,000 g for 20 min at 20 ℃. Supernatants (s) and solubilized pellets (p) were analyzed by LDS–PAGE and Coomassie Blue staining. **c**, *ex vivo* crosslinking of C-terminally H_8-tagged ρ, WT or G150D, bearing S84C and X1 (C84/C405 sensor pair) and **d**) X2 (C106/C375 sensor pair) substitutions. **e**, X1 *in vitro* crosslinking in the presence of different nucleotides. Crosslinking products were detected by LDS-PAGE and in-gel fluorescence using His-tag specific NTA-ATTO 550 stain. **f**, H_8-tagged G150D ρ forms filaments at lower concentrations than the untagged variant. Data are means ± SD. Pelleting and crosslinking experiments were performed at least three times independently with similar results. Uncropped gels are provided at the end of the Supplementary Information file. Source data are provided as a Source Data file.

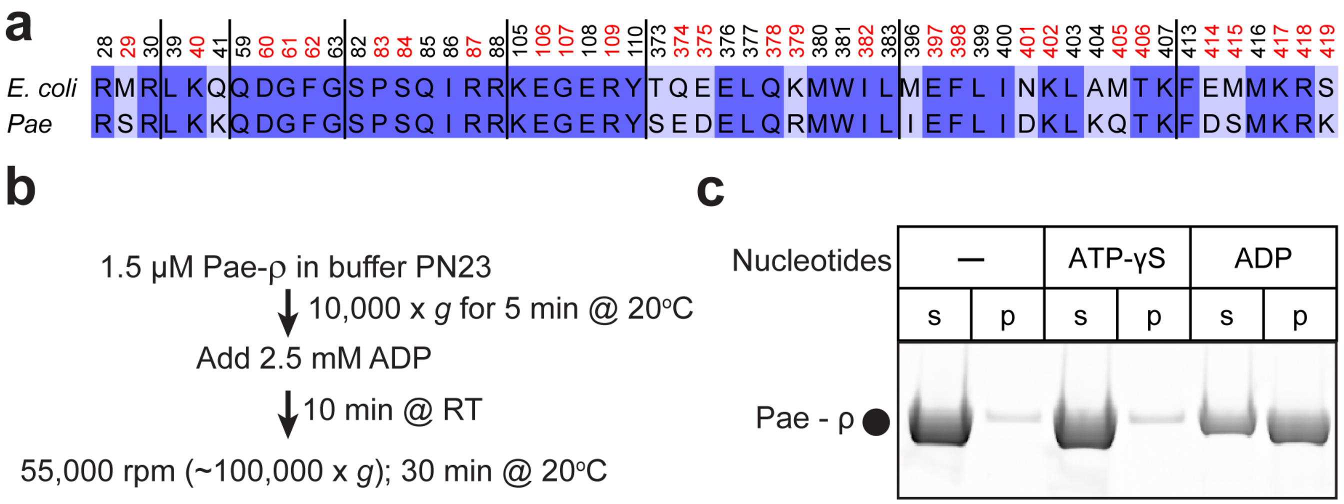

Supplementary Fig. 7. *P. aeruginosa* PAO1 (Pae) untagged ρ aggregates in the presence of ADP. a, Sequence alignment of *E. coli* and Pae ρ proteins focused on residues near the filament interface. Residues are indexed according to *E. coli* ρ; the residues directly involved in the filament formation are indicated in red. *E. coli* and Pae ρ show 81.6% identity (dark blue background) and 92.6% similarity calculated by EMBOSS Needle[1]. EMBOSS Needle uses Needleman-Wunsch algorithm and BLOSUM62 substitution matrix. **b**, Schematic illustration of Pae-ρ pelleting assay; **c**, Pae ρ pelleting pattern is similar to that observed with *E. coli* ρ (Fig. 2a and Supplementary Fig. 6b). Experiments were performed at least three times independently with similar results. Uncropped gels are provided at the end of the Supplementary Information file. Source data are provided as a Source Data file.

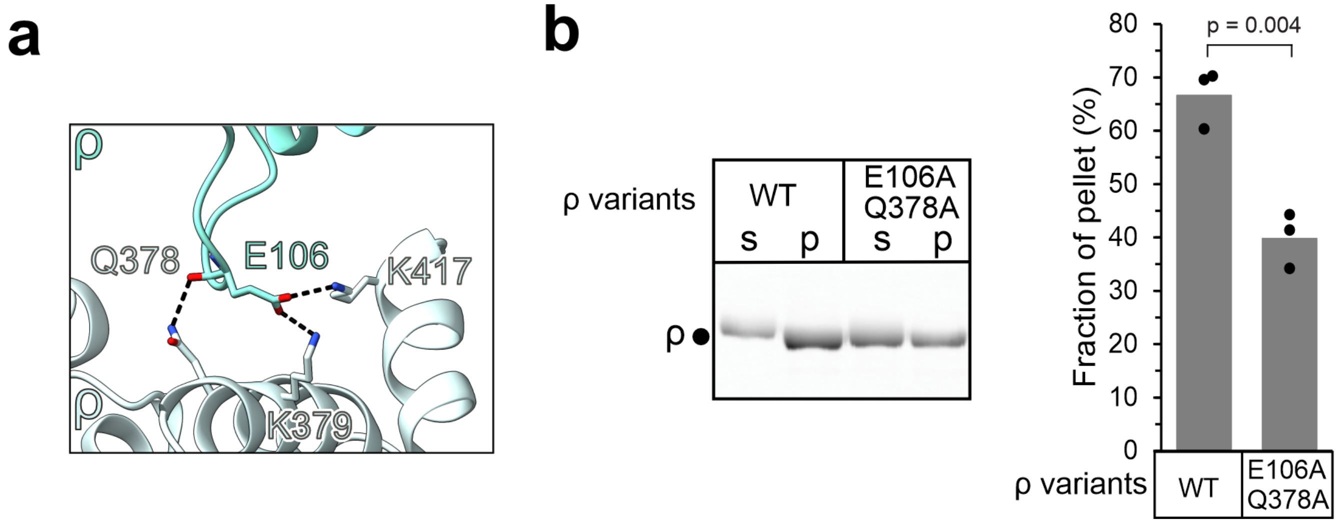

Supplementary Fig. 8. Substitutions at the filament interface compromise ρ filamentation. a, Glu106 and Gln378 residues at the filament interface make key stabilizing contacts in the G150D•H_8 filament structures. **b**, We introduced double Ala substitutions (E106A+Q378A) into the untagged ρ and compared ADP-dependent polymerization of the WT and mutant ρ proteins using pelleting assays. ρ variants (1 µM) were incubated with 3 mM ADP for 5 min at room temperature and centrifuged at ~21,100 x *g* for 10 min at 20 ℃. Left, Supernatants (s) and solubilized pellets (p) were analyzed by LDS–PAGE and Coomassie Blue staining. Right, Quantification of ρ in the pellet reveals that E106A+Q378A substitutions significantly reduce Rho pelleting (40 % as compared to 67 % for the WT ρ). Modest effects of the substitution could be expected because ρ filaments are likely stabilized by many weak interactions that cooperatively take effect as they are repeated at the multiple interfaces along the filament, making them relatively impervious to disruption. Two-tailed T-test assuming unequal variance was used to calculate p-values. Uncropped gels are provided at the end of the Supplementary Information file. Source data are provided as a Source Data file.

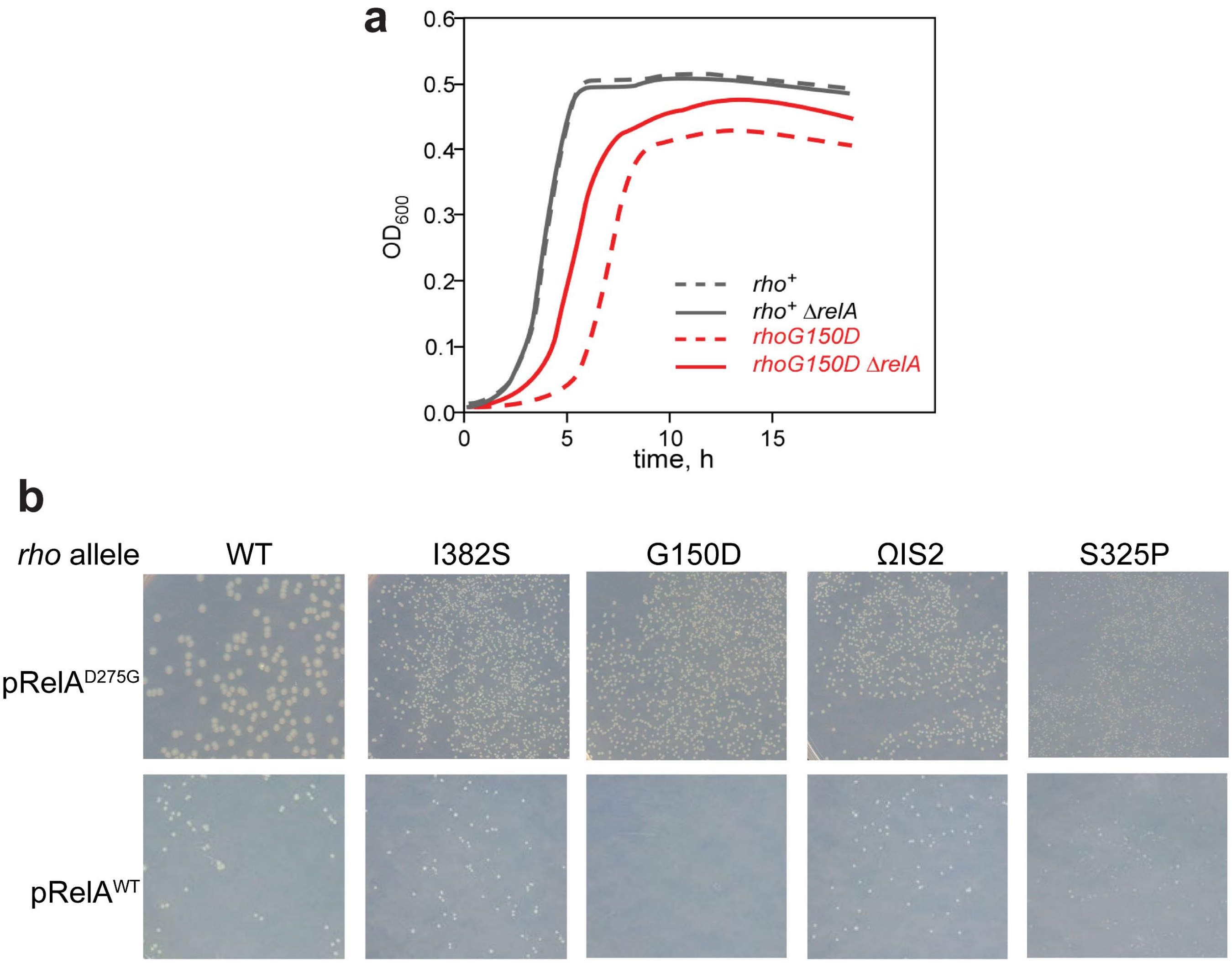

Supplementary Fig. 9. Testing effects of (p)ppGpp on G150D *rho* allele. a, Growth of WT and G150D *rho* strains carrying *relA*$^+$ or their *ΔrelA* derivatives; the *ΔrelA*::Kan was moved by P1 transduction from the Keio collection strain JW2755. Overnight cultures were diluted 1:100 into fresh MOPS EZ rich defined media (Teknova) and grown at 37 ℃ in triplicates. The growth curves were recorded with an EPOCH 2 microplate reader (BioTek). The experiment was performed three times with similar results. Source data are provided as a Source Data file. **b**, Effects of RelA expression on the growth of *E. coli* strains carrying the indicated untagged *rho* alleles. Plasmids expressing WT RelA or its catalytically-deficient D275G variant were transformed into the test strains and cells were plated onto LB plates supplemented with 100 µg/ml carbenicillin and 20 µM IPTG. Plates were incubated for 18 h (top row) or 25 h (bottom row) at 32 ℃; expression of WT RelA suppresses growth of all strains. No colonies were observed on plates with the G150D *rho* strain transformed with the plasmid expressing WT RelA. The experiment was performed four times with similar results.

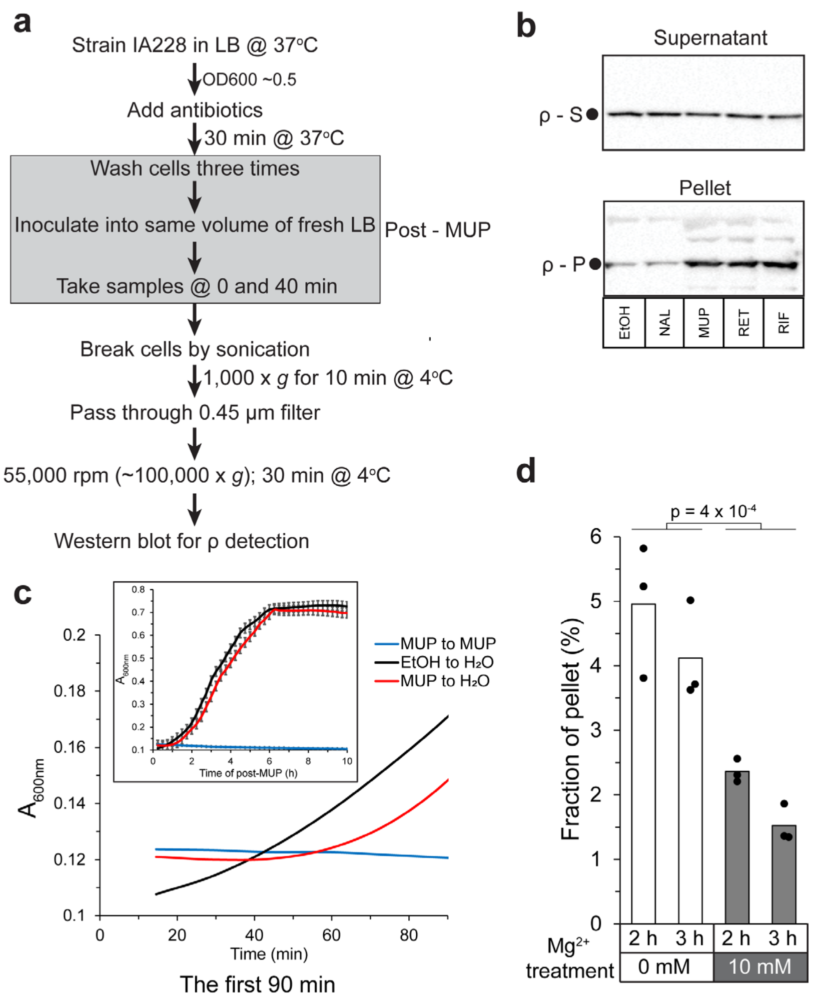

Supplementary Fig. 10. Stresses affect ρ filamentation *in vivo*. **a**, Schematic illustration of the *in vivo* pelleting assay. **b**, A representative Western blot result. After ultracentrifugation, ρ in the pellet (ρ-P) and supernatant (ρ-S; samples of the supernatant were diluted 15 times before loading) were resolved and detected by Western blot. EtOH, the same amount of ethanol was added as control; NAL, nalidixic acid; MUP, mupirocin; RET, retapamulin; RIF, rifampicin. **c,** Growth curves of the MUP recovery. A zoom-in view to show the recovery stage. The error bars are omitted for clarity. Inset, an overall view of the growth curves. MUP/EtOH treated cells were washed and reinoculated into fresh medium supplied with MUP or the same amount of autoclaved water. Cells treated with MUP were resuspended in fresh medium supplied with MUP (blue line, MUP to MUP). Cells treated with ethanol were resuspended in medium supplied with the same amount of water (black line, EtOH to H_2O). The red line (MUP to H_2O) represents the MUP-treated cells recovering in fresh medium. The experiment was performed three times with similar results. Source data are provided as a Source Data file. **d**, Cellular starvation for Mg^{2+} leads to the formation of ρ polymers. Cells were grown for 2 h and 3 h in a modified MOPS medium with either 0 mM or 10 mM $MgCl_2$. The fraction of ρ present in the pellet relative to the total amount of ρ in the sample (pellet and supernatant) is shown; see the Methods for details. The p-value was calculated between the biological replicates of 0 mM and 10 mM Mg^{2+} treatment. Two-tailed T-test assuming unequal variance was used to calculate p-value. Source data are provided as a Source Data file.

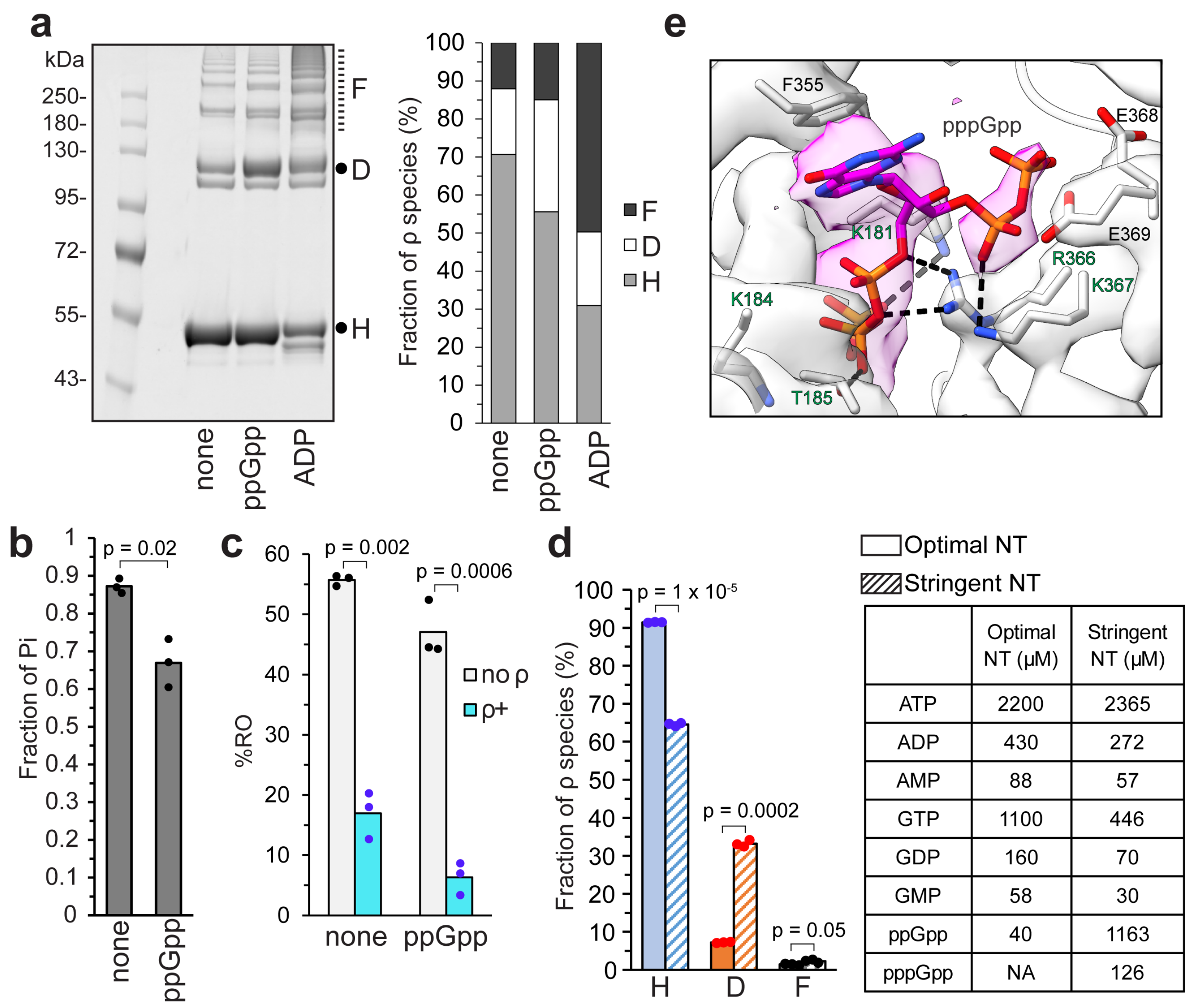

	Optimal NT (μM)	Stringent NT (μM)
ATP	2200	2365
ADP	430	272
AMP	88	57
GTP	1100	446
GDP	160	70
GMP	58	30
ppGpp	40	1163
pppGpp	NA	126

Supplementary Fig. 11. ppGpp effects on ρ oligomerization and activity. a, Crosslinking of untagged ρ-G150D^{X1} in the presence of ppGpp and ADP. Quantification of different oligomeric states is shown on the right. The experiment was performed three times with similar results. Uncropped gels are provided at the end of the Supplementary Information file. Source data are provided as a Source Data file. **b**, ppGpp modestly reduces ATPase activity of untagged WT ρ as determined by the fraction of released P_i; see Fig. 3b. Source data are provided as a Source Data file. **c**, ppGpp inhibits RNAP readthrough of the canonical λ tR1 ρ-dependent terminator in the absence of ρ and when added in complex with ρ; the experiment was performed as described in Fig. 3c. Source data are provided as a Source Data file. **d,** ppGpp induces the formation of ρ dodecamers in the presence of high concentrations of ATP. BMOE-mediated crosslinking of untagged WT ρ^{X1} was carried out in mixtures of purine nucleotides that match their experimentally determined cellular concentrations during optimal growth and stringent response induced by the addition of MUP; see main text. Two-tailed T-test assuming unequal variance was used in (**b, c, d**) to calculate p-values. Source data are provided as a Source Data file. **e**, Density of pppGpp in the nucleotide binding pocket.

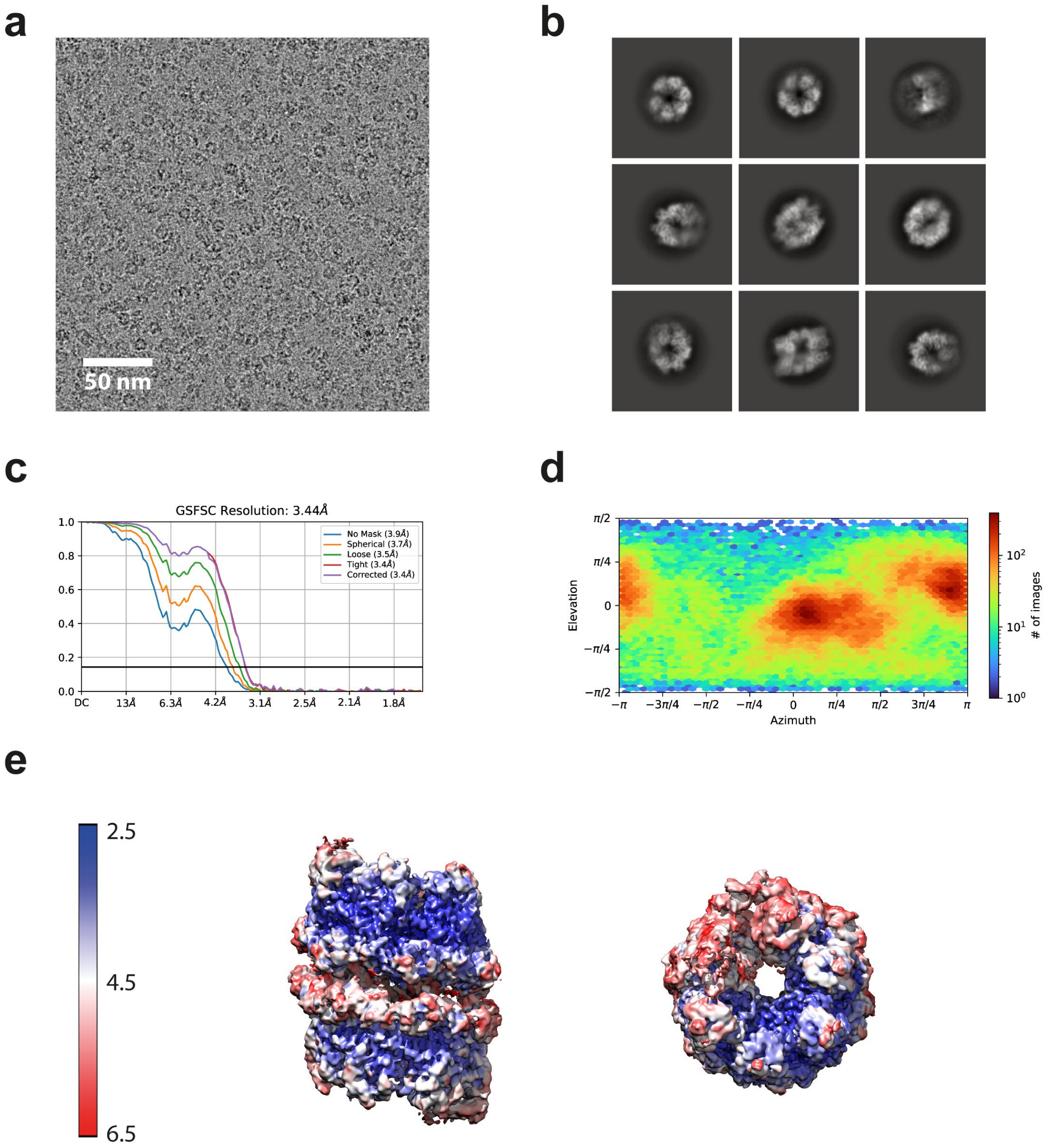

Supplementary Fig. 12. CryoEM analysis of untagged $\rho^{X1\text{-}BMOE}$. **a**, Representative cryoEM micrographs. Scale bars, 50 nm. **b**, Selected 2D class averages after reference-free 2D classification. **c**, Gold standard Fourier shell correlation analysis after helix refinement. **d**, Viewing direction distribution after NU refinement. **e**, Side (left) and top (right) views of the 3D reconstructions, colored by local resolution.

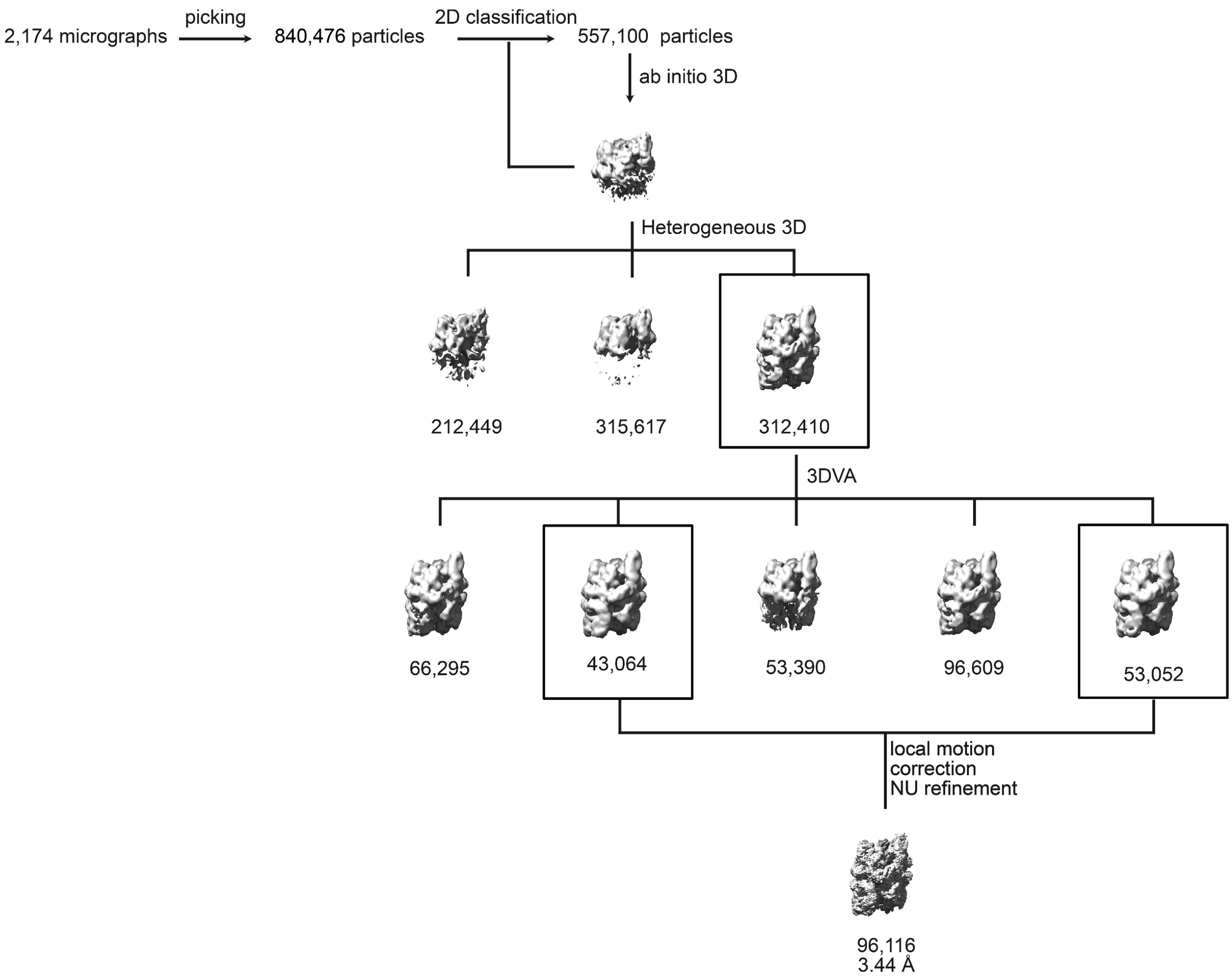

Supplementary Fig. 13. Sorting schemes of untagged $\rho^{X1\text{-}BMOE}$.

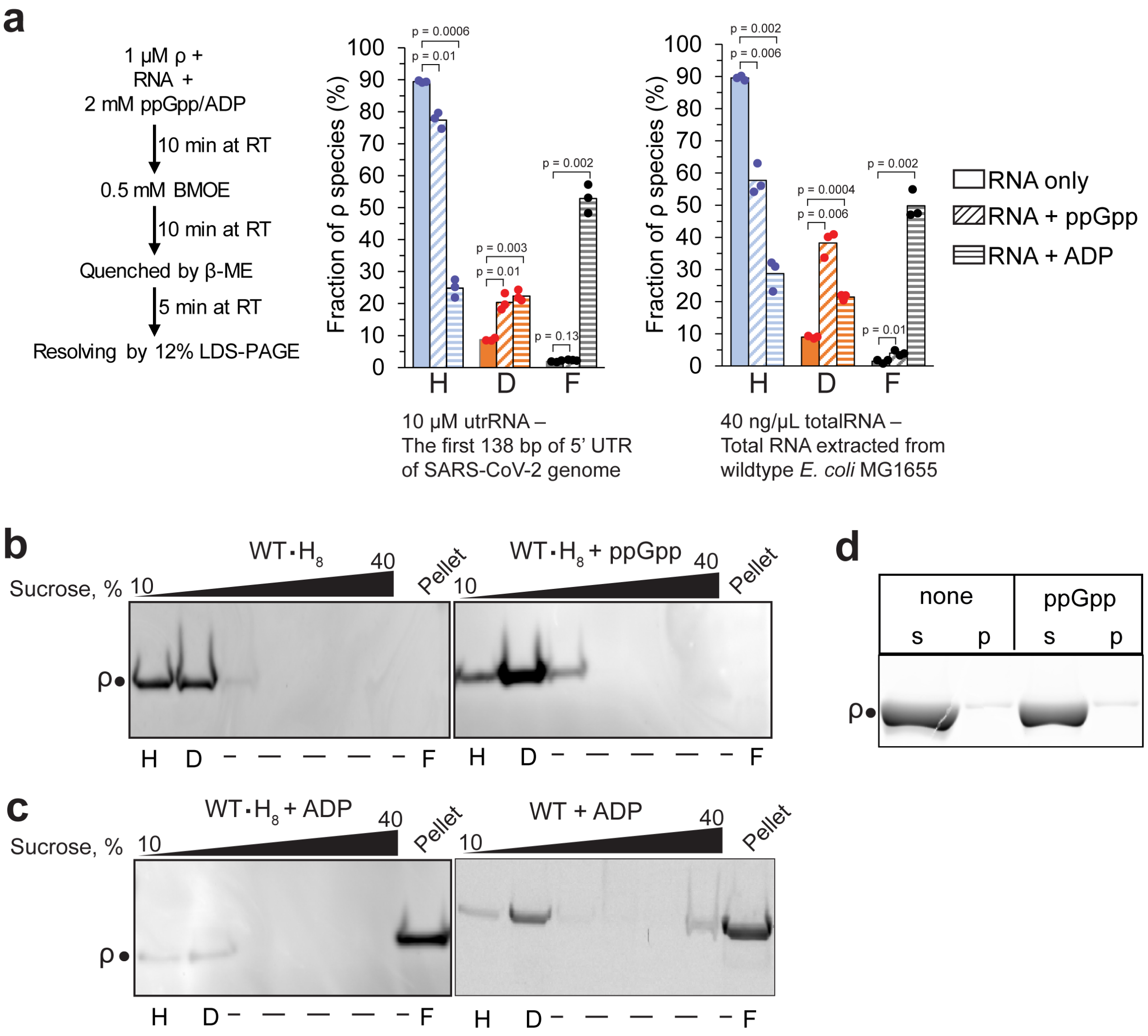

Supplementary Fig. 14. Effects of nucleotides on ρ oligomerization. a, Crosslinking of untagged ρ^{X1} in the presence of *in vitro* synthesized SARS-CoV-2 5' UTR RNA or total *E. coli* cellular RNA isolated from MG1655. Distribution of oligomeric species was determined after denaturing gel electrophoresis from three independent experiments. Two-tailed T-test assuming unequal variance was used to calculate p-values. Source data are provided as a Source Data file. **b**, Sucrose gradient centrifugation of purified His_8-tagged WT ρ following incubation with/without ppGpp. 1 µM ρ was spun down at 10,000 x *g* for 5 min at 4 ℃ to remove potential aggregates. The cleared sample was incubated with 2.5 mM of the indicated nucleotide for 10 min at room temperature. Then the reaction was loaded onto a 10 % - 40 % sucrose gradient. Ultracentrifugation of the sucrose gradient was performed at 110,000 x *g* for 16 h at 4 ℃. The resulting sucrose gradient fractions were analyzed by SurePAGE 4-12 % gel and stained with NTA-Atto 550 (Sigma). Tentative H, D, and F positions were assigned based on the sedimentation of known proteins. Experiments were performed three times independently with similar results. Source data are provided as a Source Data file. **c**, Sucrose gradient centrifugation of His_8-tagged (left) and untagged (right) WT ρ (1 µM) preincubated with 2.5 mM ADP. The experiment was performed as in panel b except that Coomassie blue staining (GelCode Blue Stain; ThermoFisher) was used to visualize untagged ρ. Experiments were performed three times independently with similar results. Source data are provided as a Source Data file. **d**, ppGpp alone does not promote ρ pelleting. 1 µM untagged WT ρ preincubated with/without 2 mM ppGpp was spun down at ~280,000 x *g* for 20 min at 20 ℃. Supernatants (s) and solubilized pellets (p) were analyzed by LDS–PAGE and Coomassie Blue staining. Experiments were performed three times independently with similar results. Source data are provided as a Source Data file. Uncropped gels are provided at the end of the Supplementary Information file.

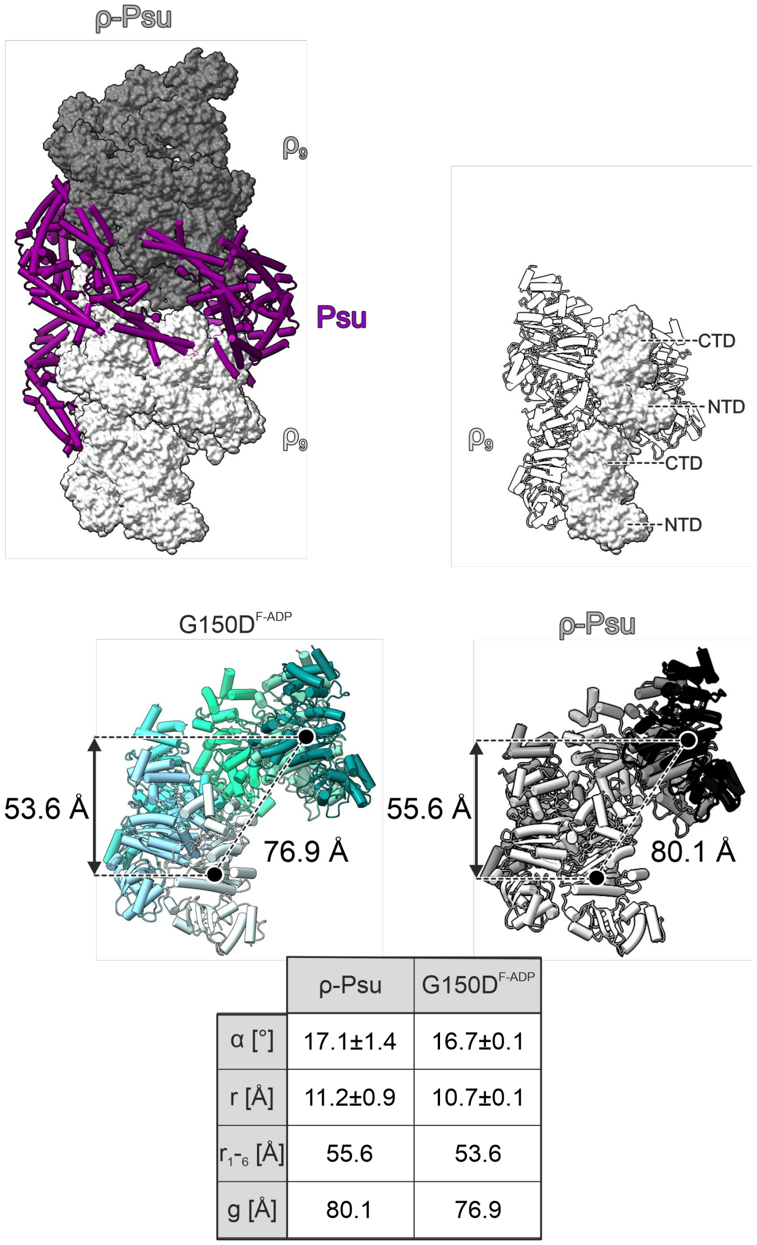

	ρ-Psu	$G150D^{F\text{-}ADP}$
α [°]	17.1±1.4	16.7±0.1
r [Å]	11.2±0.9	10.7±0.1
r_{1}-$_{6}$ [Å]	55.6	53.6
g [Å]	80.1	76.9

Supplementary Fig. 15. Comparison of Psu-mediated ρ oligomers with ρ filaments. Left, multiple Psu dimers bridge two individual ρ rings (PDB ID: 8PEW; grey and white). Right, each Rho can assemble into a nonamer ring with a similar helical geometry as observed in the ρ filament structure (see also Fig. 5g). Bottom, hexamers extracted from the filament structure (left) and from a ρ nonamer (right). Helical parameters are calculated as described in Fig. 1d.

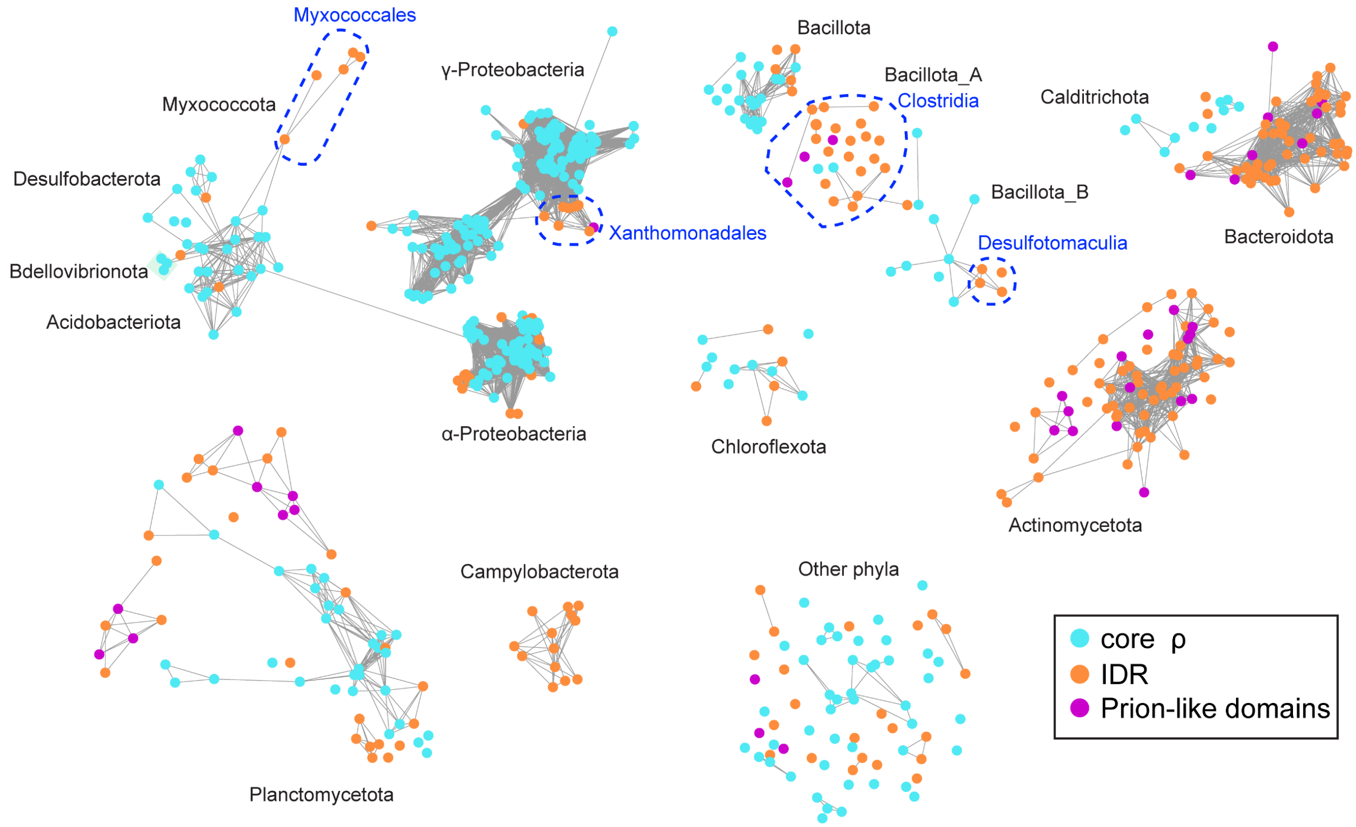

Supplementary Fig. 16. ρ proteins were grouped by the percentage of identity (%id). Nodes represent ρ proteins, and a pair of nodes are connected by an edge (grey line) if %id > 75 %; hypothetical IDRs (intrinsically disordered regions) and prion-like domains are indicated (See Methods for the construction of this network).

Supplementary Table 1. Plasmids and strains used in this study.

Plasmids	Key features	Source
pET24-ρ	Untagged wild type ρ under T7 promoter	[2]
pIA1301	T7 promoter- ρ-His_6	This study
pIA1307	T7 promoter- ρ [G150D]-His_8	This study
pIA1538	T7 promoter - [G150D]	This study
pIA1510	T7 promoter- ρ [S84C]-His_8	This study
pIA1511	T7 promoter- ρ [G150D S84C]-His_8	This study
pIA1513	T7 promoter- ρ [E106C E375C]-His_8	This study
pIA1514	T7 promoter- ρ [S84C M405C]-His_8	This study
pIA1515	T7 promoter- ρ [G150D S84C M405C]-His_8	This study
pIA1516	T7 promoter- ρ [G150D E106C E375C]-His_8	This study
pIA1539	T7 promoter- ρ [M405C]	This study
pIA1540	T7 promoter- ρ [S84C M405C]	This study
pIA1686	T7 promoter- ρ [E106A Q378A]	This study
pIA1634	T7 promoter-His_{10}-SUMO-*P. aeruginosa* ρ	This study
pR1-1His	Wild type RelA under IPTG-inducible promoter	[3]
pR1-1His(D275G)	Inactive RelA mutant under IPTG-inducible promoter	[3]
pIA267	λ PR promoter – C-less A26 ITR - λ tR1 terminator template	[4]
**Primers**	**Sequence (5' – 3')**	**Source**
λPR_UP	CGTTAAATCTATCACCGCAAGG	This study
λPR_DN	CAGTTCCCTACTCTCGCATG	This study
Rut_UP	CCTCACTAATACGACTCACTATAGGGATCCAGATCCCGGAA	This study
Rut_DN	AGCCTCGTTGCGTTTGTTT	This study
**Strains**	**Genotype**	**Source**
IA228	MG1655* Δ*rfaH*	[5]
IA305	MG1655* Δ*rfaH* Δ*rac rho*G150D	[5]
IA306	MG1655* Δ*rfaH* Δ*rac rhoL*-ΩIS2-*rho*	[5]
IA539	MG1655* Δ*rfaH* Δ*rac*	This study
IA791	MG1655* Δ*rfaH* Δ*rac rho*G150D *relA*::Kn	This study
IA793	MG1655 Δ*rfaH* Δ*rac relA*::Kn	This study
$ppGpp^0$	MG1655 Δ*relA* Δ*spoT*	Christophe Herman (Baylor College of Medicine)

*, Δ*rfaH* strains were subsequently found to have mutations absent in the reference MG1655 strain, see [6] for details.

Supplementary Table 2. CryoEM data collection, refinement, and validation statistics.

Data collection and processing					
	ρ^{G150D}	ρ^{G152D}	ρ^{pppGpp}	ρ^{ADP}	$\rho^{X1\text{-}BMOE}$
Microscope	FEI Titan Krios G3i				
Voltage [keV]	300				
Camera	Falcon 3EC				
Magnification (nominal)	96,000x				
Pixel size at detector [Å/pixel]	0.832				
Total electron exposure [e^-/Å^2]	42				
Exposure rate [e^-/pixel/s]	0.7				
Frames collected during exposure	33				
Defocus range [µm]	0.8 - 2				
Automation software	EPU version 2.10				
Micrographs					
Collected	2511	1642	1477	1599	2218
Used	2511	1578	1467	1544	2174
Particle images					
Total extracted	495,704	428,112	641,595	602,082	840,476
Final	140,359	222,106	200,796	314,323	96,116
Point-group or helical symmetry parameters	C1	C1	C1	C1	C1
Resolution [Å]					
Global					
$FSC_{0.143}$[(a)]	3.9 / 3.3	3.8 / 3.3	3.5 / 3.0	3.1 / 2.6	3.9 / 3.4
(unmasked/masked)	1.8 - 39	1.8 - 30	2.2 - 35	1.8 - 35	2.6 - 43
Local resolution range [Å^2]					
Map sharpening *B* factor [Å^2]	-87.6	-92.7	-101.7	-97.6	-85.4
Map sharpening methods	local B-factor				
Refinement software					
Package	PHENIX version 1.20_4459				
Routine	real.space.refine				
**Model composition**					
	ρ^{G150D}	ρ^{G152D}	ρ^{pppGpp}	ρ^{ADP}	$\rho^{X1\text{-}BMOE}$
Model composition					
Non-H atoms	59.922	59.922	19.9007	19.912	39.552
Protein residues	7.542	7.542	2.508	2.508	5.016
RNA residues	-	-	-	-	-
Mg^{2+} ions	18	18	-1	4	4
ADP	18	18	-	6	-
BeF	-	-	-	-	-
pppGpp	-	-	4	-	-
ME7 (BMOE)					5
**Model Refinement**					
Model-Map scores					
CC[(b)] (mask)	0.87	0.87	0.82	0.85	85
CC (volume)	0.87	0.87	0.82	0.85	84
Average grouped B factors [Å^2]					
Overall	124	116	106	117	163
Protein	124	116	106	117	163
Mg^{2+} ions	134	126	37	70	104
ADP	131	121	-	134	-
pppGpp	-	-	99	-	-
BMOE	-	-	-	-	182
Rmsd[(c)] from ideal values					
Bond lengths [Å]	0.002	0.001	0.005	0.004	0.002
Bond angles [°]	0.436	0.443	0.625	0.688	0.393
**Validation[(d)]**					
MolProbity score	1.48	1.45	1.62	1.52	1.47
CaBLAM outliers [%]	0.48	0.72	0.89	0.97	0.91
Clashscore	5.65	6.15	12.97	9.72	8.80
Poor rotamers [%]	1.67	1.39	0.33	1.02	0.00
Cβ deviations	0.0	0.0	0.0	0.0	0.0
EMRinger score	2.94	2.39	1.71	2.21	1.64
Ramachandran plot					

Favored [%]<br>Allowed [%]<br>Outliers [%]	98.80<br>1.20<br>0.0	98.80<br>1.20<br>0.0	98.12<br>1.88<br>0.00	98.56<br>1.44<br>0.0	99.16<br>0.84<br>0.0
Ramachandran *Z*-score (rmsd)					
Overall	1.36 (0.10)	1.76 (0.10)	0.89 (0.17)	0.85 (0.17)	1.12 (0.12)
Helices	1.19 (0.09)	1.26 (0.09)	1.31 (0.16)	1.14 (0.16)	0.33 (0.12)
Sheets	0.76 (0.15)	1.39 (0.14)	0.12 (0.26)	0.86 (0.28)	1.37 (0.25)
Loops	0.90 (0.11)	0.17 (0.11)	0.03 (0.20)	-0.11 (0.19)	1.26 (0.13)
Data deposition					
Reconstruction (EMDB)	EMD-18132	EMD-18133	EMD-18131	EMD-18130	EMD-50352
Coordinates (PDB)	8Q3P	8Q3Q	8Q3O	8Q3N	9FF7

[a] FSC, Fourier shell correlation
[b] CC, correlation coefficient
[c] Rmsd, root-mean-square deviation
[d] Using MolProbity[5].

Supplementary References:

1 Madeira, F. *et al.* The EMBL-EBI Job Dispatcher sequence analysis tools framework in 2024. *Nucleic Acids Res* **52**, W521-w525 (2024). https://doi.org:10.1093/nar/gkae241

2 Skordalakes, E. & Berger, J. M. Structure of the Rho transcription terminator: mechanism of mRNA recognition and helicase loading. *Cell* **114**, 135-146 (2003).

3 Wang, B. *et al.* Affinity-based capture and identification of protein effectors of the growth regulator ppGpp. *Nat Chem Biol* **15**, 141-150 (2019). https://doi.org:10.1038/s41589-018-0183-4

4 Artsimovitch, I. & Landick, R. The transcriptional regulator RfaH stimulates RNA chain synthesis after recruitment to elongation complexes by the exposed nontemplate DNA strand. *Cell* **109**, 193-203 (2002). https://doi.org:10.1016/s0092-8674(02)00724-9

5 Hu, K. & Artsimovitch, I. A Screen for rfaH Suppressors Reveals a Key Role for a Connector Region of Termination Factor Rho. *MBio* **8**, e00753-00717 (2017). https://doi.org:10.1128/mBio.00753-17

6 Said, N. *et al.* Sm-like protein Rof inhibits transcription termination factor rho by binding site obstruction and conformational insulation. *Nat Commun* **15**, 3186 (2024). https://doi.org:10.1038/s41467-024-47439-6

Source Data

Supplementary Fig. 6b

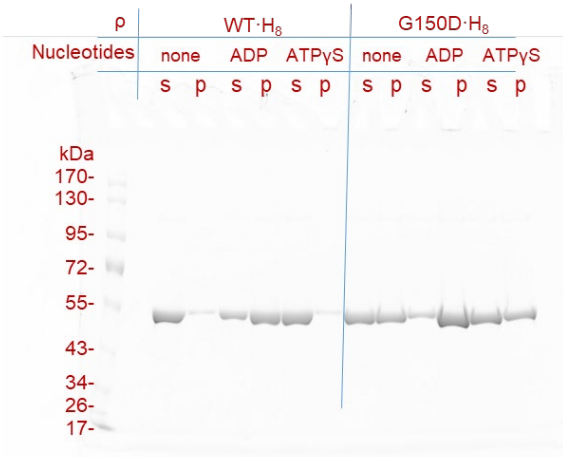

Supplementary Fig. 6c

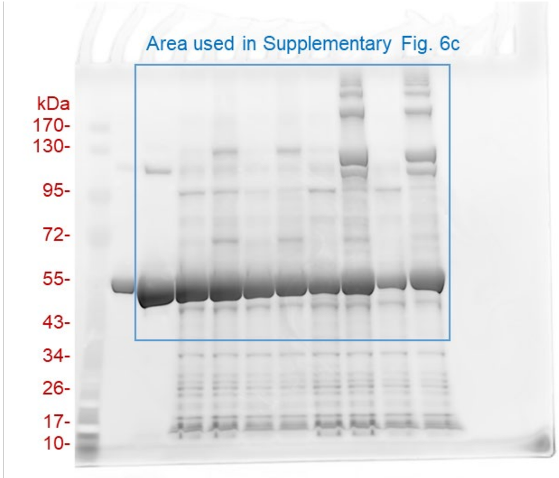

Supplementary Fig. 6d

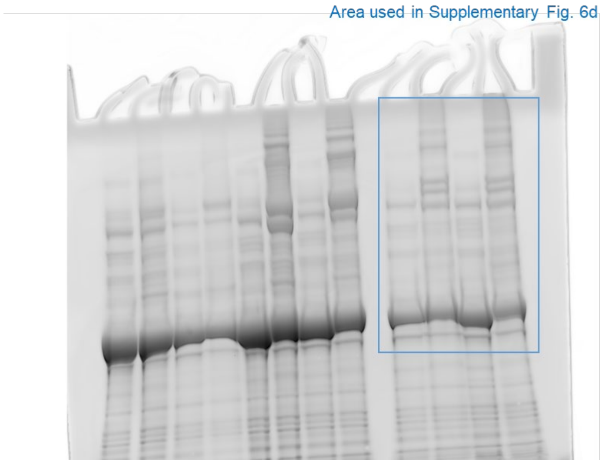

Supplementary Fig. 6e

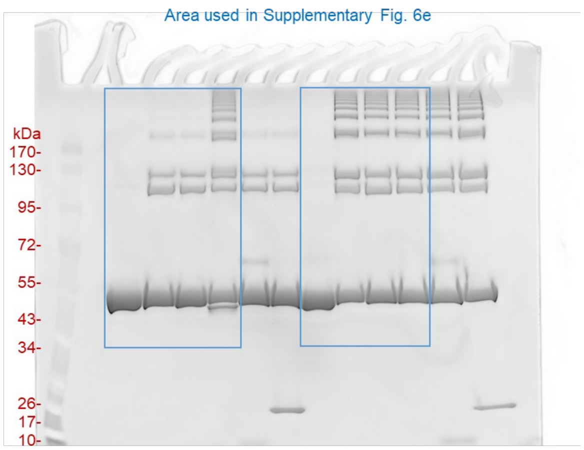

Supplementary Fig. 6f

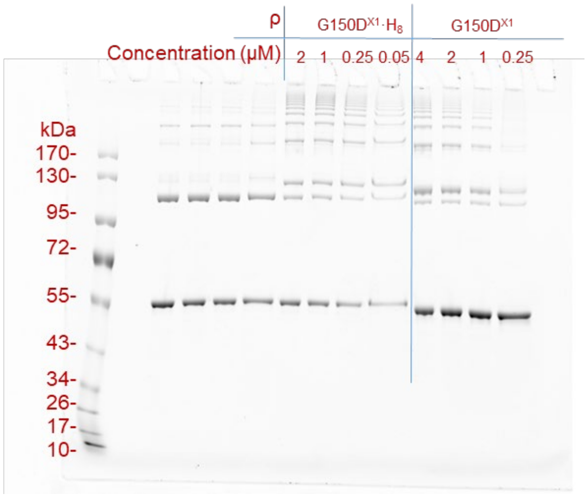

Supplementary Fig. 7c

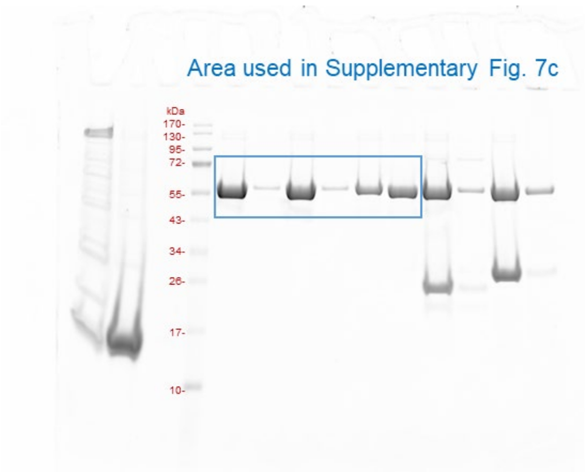

Supplementary Fig. 8b

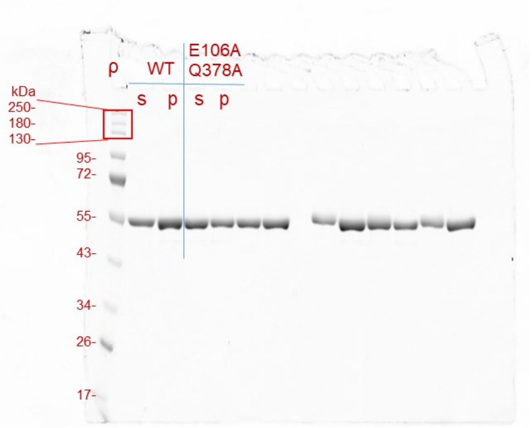

Supplementary Fig. 11a

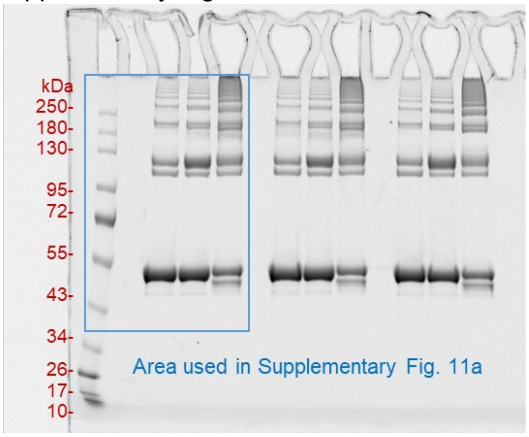

Supplementary Fig. 14b and c

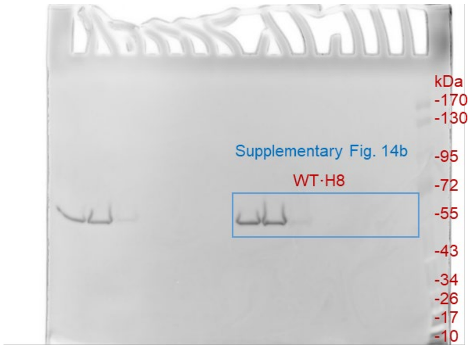

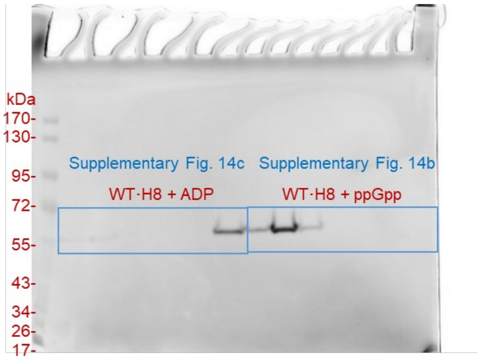

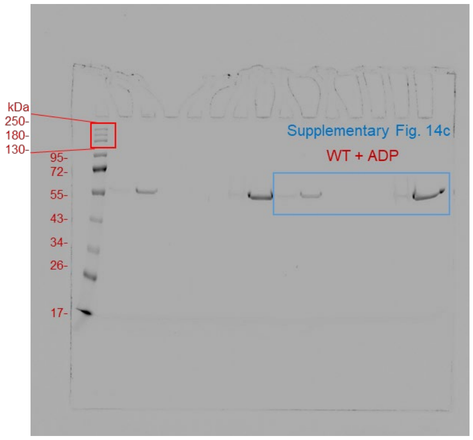

Supplementary Fig. 14d

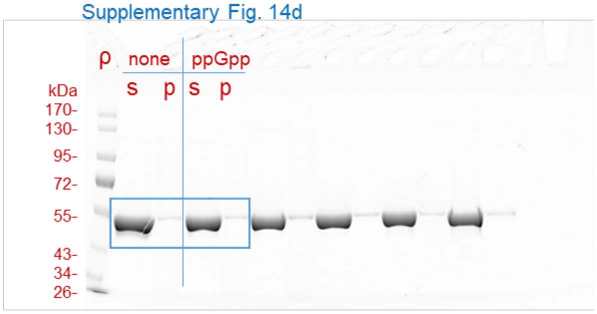

Supplement: Supplementary file 1 — Supplementary Information [file 41467_2025_56824_MOESM1_ESM.pdf]
