# Supplementary material for: Nucleotide-induced hyper-oligomerization inactivates transcription termination factor ρ
Source: Nat Commun. 2025 Feb 15;16:1653. doi: 10.1038/s41467-025-56824-8 (PMC11829017; doi:10.1038/s41467-025-56824-8)
Supplement: Supplementary file 3 — Description of Additional Supplementary Files [file 41467_2025_56824_MOESM3_ESM.pdf]

Description of Additional Supplementary Files

Supplementary Data 1. ρ conservation and IDR/prion-like domain analysis. a, The successfully downloaded genomes and identified ρ sequences in our representative database. b, Result of IDR/prion-like domain detection for ρ sequences in our representative database. Y, presence. N, absence.
