# Supplementary material for: Nucleotide-induced hyper-oligomerization inactivates transcription termination factor ρ
Source: Nat Commun. 2025 Feb 15;16:1653. doi: 10.1038/s41467-025-56824-8 (PMC11829017; doi:10.1038/s41467-025-56824-8)
Supplement: Supplementary file 5 — Reporting Summary [file 41467_2025_56824_MOESM5_ESM.pdf]

nature portfolio

Corresponding author(s): Irina Artsimovitch

Last updated by author(s): Jan 10, 2025

Reporting Summary

Nature Portfolio wishes to improve the reproducibility of the work that we publish. This form provides structure for consistency and transparency in reporting. For further information on Nature Portfolio policies, see our Editorial Policies and the Editorial Policy Checklist.

Statistics

For all statistical analyses, confirm that the following items are present in the figure legend, table legend, main text, or Methods section.

n/a	Confirmed	
☐	☒	The exact sample size (n) for each experimental group/condition, given as a discrete number and unit of measurement
☐	☒	A statement on whether measurements were taken from distinct samples or whether the same sample was measured repeatedly
☐	☒	The statistical test(s) used AND whether they are one- or two-sided<br>*Only common tests should be described solely by name; describe more complex techniques in the Methods section.*
☒	☐	A description of all covariates tested
☒	☐	A description of any assumptions or corrections, such as tests of normality and adjustment for multiple comparisons
☐	☒	A full description of the statistical parameters including central tendency (e.g. means) or other basic estimates (e.g. regression coefficient) AND variation (e.g. standard deviation) or associated estimates of uncertainty (e.g. confidence intervals)
☐	☒	For null hypothesis testing, the test statistic (e.g. F, t, r) with confidence intervals, effect sizes, degrees of freedom and P value noted<br>*Give P values as exact values whenever suitable.*
☒	☐	For Bayesian analysis, information on the choice of priors and Markov chain Monte Carlo settings
☒	☐	For hierarchical and complex designs, identification of the appropriate level for tests and full reporting of outcomes
☒	☐	Estimates of effect sizes (e.g. Cohen's d, Pearson's r), indicating how they were calculated

Our web collection on statistics for biologists contains articles on many of the points above.

Software and code

Policy information about availability of computer code

Data collection	EPU, version 2.14 (cryoEM/SPA data acquisition; Thermo Fisher Scientific; referenced in text)<br>Image Lab, version 6.1 (Western Blot data collection and quantification; BioRad; referenced in text)<br>FL solutions, version 7.1 (Tryptophan fluorescence data collection; Hitachi; referenced in text)<br>Gen5, version 3.12 (growth curves acquisition; BioTek; referenced in text)<br>Amersham Typhoon, version 3.0.0.2 (Data collection for SDS-PAGE gel based experiments; Cytiva; referenced in text)
Data analysis	ChimeraX, version 1.4 (cryoEM reconstructions figure preparation; referenced in text)<br>Chimera, version 1.16 (cryoEM reconstructions figure preparation)<br>Coot, version 0.9.6 (model building; referenced in text)<br>cryoSPARC, version 3.2.2 (all cryoEM image analysis; referenced in text)<br>ImageQuant, version 5.2 (quantification of bands on gels; Cytiva; referenced in text)<br>Molprobity, version 4.5.1 (structure evaluation; referenced in text)<br>LigPlot+, version 2.2.8 (used for the visualization and analysis of protein-protein/ligand interactions)<br>PHENIX, version 1.20_4459 (real space refinement; referenced in text)<br>PyMOL, version 2.4.0 (structure figure preparation; referenced in text)<br>Microsoft EXCEL, version 2307 (statistics analysis; referenced in text)<br>hmmsearch, version 3.3 (identifying ρ sequences; referenced in text)<br>Dialign, version 2.2.1 (multiple sequence alignment; referenced in text)<br>Jalview, version 2.11.2.7 (multiple sequence alignment editing and visulization; referenced in text)

WebLogo, version 3.7.8 (sequence logo generation; referenced in text)
BLAST+, version 2.9.0 (all versus all search; flagellar export ATPase FliI sequences identification; referenced in text)
Cytoscape, version 3.9.1 (visualization of ρ similarity network; referenced in text)
clusterMaker2, version 2.3.2 (Markov clustering analysis of ρ sequences; referenced in text)
metapredict, version 2.6 (Intrinsically disordered regions prediction; referenced in text)
PrionW (Prion-like domain prediction; referenced in text)
R, version 4.2.2 (Phylogenetic tree editing and visualization; referenced in text)
AlphaFold3 (referenced in text)

For manuscripts utilizing custom algorithms or software that are central to the research but not yet described in published literature, software must be made available to editors and reviewers. We strongly encourage code deposition in a community repository (e.g. GitHub). See the Nature Portfolio guidelines for submitting code & software for further information.

Data

Policy information about availability of data

All manuscripts must include a data availability statement. This statement should provide the following information, where applicable:
- Accession codes, unique identifiers, or web links for publicly available datasets
- A description of any restrictions on data availability
- For clinical datasets or third party data, please ensure that the statement adheres to our policy

CryoEM reconstructions have been deposited in the Electron Microscopy Data Bank (https://www.ebi.ac.uk/pdbe/emdb) under accession codes EMD-18132 (https://www.ebi.ac.uk/pdbe/entry/emdb/EMD-18132; ρG150D), EMD-18133 (https://www.ebi.ac.uk/pdbe/entry/emdb/EMD-18133; ρG152D), EMD-18131 (https://www.ebi.ac.uk/pdbe/entry/emdb/EMD-18131; ρ-pppGpp), EMD-18130 (https://www.ebi.ac.uk/pdbe/entry/emdb/EMD-18130; ρ-ADP complex), and EMD-50352 (https://www.ebi.ac.uk/pdbe/entry/emdb/EMD-50352; ρX1-BMOE complex). Structure coordinates have been deposited in the RCSB Protein Data Bank (https://www.rcsb.org) with accession codes 8Q3P (https://www.rcsb.org/structure/8Q3P ρG150D), 8Q3Q (https://www.rcsb.org/structure/8Q3Q; ρG152D), 8Q3O (https://www.rcsb.org/structure/8Q3O; ρ-pppGpp), 8Q3N (https://www.rcsb.org/structure/8Q3N; ρ-ADP) and 9FF7 (https://www.rcsb.org/structure/9FF7; ρX1-BMOE complex). All other data are contained in the manuscript or the Supplementary Information. Source data are provided with this paper. Structure coordinates used in this study are available from the RCSB Protein Data Bank (https://www.rcsb.org) under accession codes 6WA8 (https://www.rcsb.org/structure/6WA8) and 8PEW (https://www.rcsb.org/structure/8PEW).

Human research participants

Policy information about studies involving human research participants and Sex and Gender in Research.

Reporting on sex and gender	N/A
Population characteristics	N/A
Recruitment	N/A
Ethics oversight	N/A

Note that full information on the approval of the study protocol must also be provided in the manuscript.

Field-specific reporting

Please select the one below that is the best fit for your research. If you are not sure, read the appropriate sections before making your selection.

[x] Life sciences [ ] Behavioural & social sciences [ ] Ecological, evolutionary & environmental sciences

For a reference copy of the document with all sections, see nature.com/documents/nr-reporting-summary-flat.pdf

Life sciences study design

All studies must disclose on these points even when the disclosure is negative.

Sample size	Sample sizes are described in the Methods. The sample sizes are standard for the in vitro assays performed in the study.<br>For cryoEM analysis, the sample size was chosen so as to yield a large number of particle images on the grids while avoiding non-specific aggregation.<br>For biochemical and biophysical assays, sample sizes were chosen so as to provide significant and reproducible signals (nucleotide binding) or to provide clearly visible and quantifiable bands on gels (SDS PAGE analyses).
Data exclusions	Structural analysis by cryoEM involved the sorting of high quality particle images and rejection of poor quality particle images. Poor quality particle images may be due to compositional heterogeneity in the sample or may originate from particles being damaged in the process of grid preparation, e.g. at the air-water interface.<br>For other experiments, no data were excluded from the analyses.

Replication	The numbers of technical and biological replicates are indicated in the text and figure legends.
Randomization	This study reports results from genetic/biochemical/biophysical experiments with bacterial proteins. Randomization was not required for this study, as (1) no human or animal subjects were studied, (2) quantitative data were collected, (3) no subjective interpretations were required and (4) there was no danger of confounding independent variables in the experimental design.
Blinding	N/A; the experiments did not involve human or animal subjects, and as the results from the experiments can be objectively evaluated/ quantified.

Reporting for specific materials, systems and methods

We require information from authors about some types of materials, experimental systems and methods used in many studies. Here, indicate whether each material, system or method listed is relevant to your study. If you are not sure if a list item applies to your research, read the appropriate section before selecting a response.

Materials & experimental systems

n/a	Involved in the study	
☐	☒	Antibodies
☒	☐	Eukaryotic cell lines
☒	☐	Palaeontology and archaeology
☒	☐	Animals and other organisms
☒	☐	Clinical data
☒	☐	Dual use research of concern

Methods

n/a	Involved in the study	
☒	☐	ChIP-seq
☒	☐	Flow cytometry
☒	☐	MRI-based neuroimaging

Antibodies

Antibodies used	Polyclonal anti-Rho rabbit/IgG antibody, a gift from Evgeny Nudler (NUY); diluted 1:10000
Validation	Tested for recognition of purified Rho proteins in vitro and mutant Rho proteins in E. coli; published in PMID: 28559482
